# Active Brownian Filamentous Polymers under Shear Flow

**DOI:** 10.3390/polym10080837

**Published:** 2018-07-30

**Authors:** Aitor Martín-Gómez, Gerhard Gompper, Roland G. Winkler

**Affiliations:** Theoretical Soft Matter and Biophysics, Institute of Complex Systems and Institute for Advanced Simulation, Forschungszentrum Jülich, D-52425 Jülich, Germany; a.gomez@fz-juelich.de (A.M.-G.); g.gompper@fz-juelich.de (G.G.)

**Keywords:** semiflexible polymer, active Brownian particle, active polymer, polymer conformations, polymer dynamics, colored noise, viscosity, rheology

## Abstract

The conformational and rheological properties of active filaments/polymers exposed to shear flow are studied analytically. Using the continuous Gaussian semiflexible polymer model extended by the activity, we derive analytical expressions for the dependence of the deformation, orientation, relaxation times, and viscosity on the persistence length, shear rate, and activity. The model yields a Weissenberg-number dependent shear-induced deformation, alignment, and shear thinning behavior, similarly to the passive counterpart. Thereby, the model shows an intimate coupling between activity and shear flow. As a consequence, activity enhances the shear-induced polymer deformation for flexible polymers. For semiflexible polymers/filaments, a nonmonotonic deformation is obtained because of the activity-induced shrinkage at moderate and swelling at large activities. Independent of stiffness, activity-induced swelling facilitates and enhances alignment and shear thinning compared to a passive polymer. In the asymptotic limit of large activities, a polymer length- and stiffness-independent behavior is obtained, with universal shear-rate dependencies for the conformations, dynamics, and rheology.

## 1. Introduction

Active matter is composed of agents which either convert internal energy or exploit energy from the environment to generate directed motion [1,2,3,4,5,6,7]. The associated out-of-equilibrium character of active matter is the origin of a number of fascinating phenomena, such as activity-driven phase separation or large-scale collective motion [1,2,3,4,5,6,7,8,9,10,11,12,13]. On the nano- and microscale, biology provides a plethora of active agents ranging from enzymes [14,15] and the cytoskeleton in living cells [1,3,16,17,18,19,20,21,22] to sperm, algae, bacteria, and a diversity of other planktonic microorganisms [1,4,23,24]. Furthermore, artificial active particles have been synthesized utilizing various concepts [5,25,26,27,28,29]. Thereby, active agents exhibit a variety of forms and shapes—from (near) spherical (*Chlamydomonas reinhardtii*, *Volvox*) to cylindrical (*Proteus mirabilis* [30], self-assembled dinoflagellates [31,32]), and filamentous, polymer-like structures (actin filaments, microtubules, linear assemblies of Janus particles [33,34]). In fact, active systems with internal degrees of freedom, such as linear chains [7,34,35,36,37,38,39,40,41,42,43,44,45,46,47,48,49,50,51,52,53,54] or other forms of organization [33,34,55,56], denoted as “active colloidal molecules” in Ref. [34], are particularly interesting and give rise to novel conformational [39,41,47,52], dynamical [45,53,57,58,59], and collective phenomena [12,16,56,60,61,62,63,64,65,66]. Examples range from activity-induced polymer swelling and shrinkage [7,39,41,47,52], enhanced diffusive motion and dynamics [40,41,53,59]—as observed in microtubuli [67], actin filaments [68], chromosomal loci in simple organisms [69,70], or in the chromatin dynamics in eukaryotes [71]—to mesoscale turbulence [12,56] or streaming nematics [60,65].

In theoretical and simulation studies, filamentous, polymer-like active agents are typically described as semiflexible polymers composed of monomers, which are propelled by an active force. The properties of the active force depend on the system of interest. Actin filaments or microtubules in motility assays or motor-protein carpets [61] are driven by tangential forces with respect to the filament contour [35,43,45,66]. Alternatively, polymers comprised of active Brownian particles (ABPs) are driven by active forces, which change their propulsion direction independently in a diffusive manner [5,10,47,52,72]. So far, no polymer-like assembly of ABPs has been synthesized. However, activity can also be considered as an external random colored-noise force experienced by the polymer, as discussed in Refs. [4,6,51,52,53]. In this context, “activity” is an extension of the theoretical description toward more complex out-of-equilibrium environments, which break detailed balance and the fluctuation–dissipation theorem [73].

In this article, the conformational and rheological properties of an active polymer subject to colored noise and exposed to shear flow are studied. In particular, the interplay between activity and shear flow is investigated. The effect of propulsion on the rheology of entangled, isotropic solutions of tangentially driven semiflexible polymers has been addressed in Ref. [35], and an accelerated relaxation has be found at long times, resulting in a reduced low-frequency viscosity. Specifically, the transport of microorganisms and their assemblies, which is omnipresent in nature, e.g., plankton in aquatic environments and microfluidic devices, is strongly affected by fluid flow [74]. In such habitats, shear flow is pervasive and determines the destination of microorganisms. Our goal is to unravel the properties of semiflexible filamentous polymers in such an out-of-equilibrium environment induced by both colored noise and fluid flow. Our studies reveal an intimate coupling of activity and shear flow, which leads to distinct differences in the non-equilibrium polymer conformations, dynamics, and rheology. Specifically, shear-rate dependent power-laws are modified at large activities. It is noteworthy that polymer-length- and stiffness-independent universal dependencies on the shear rate are predicted in the asymptotic limit of large activities.

The article is organized as follows. Section 2 describes the model of the active polymer in shear flow and presents the equations of motion and their solution. In Section 3, results for the shear-rate dependence of the relaxation times and conformational properties are presented for various activities and stiffness. The viscosity of the polymer is considered in Section 4. Section 5 discusses the various findings, and Section 6 summarizes the major results of our study.

## 2. Model: Active Brownian Filament/Polymer

### 2.1. Equation of Motion

**Figure 1:**
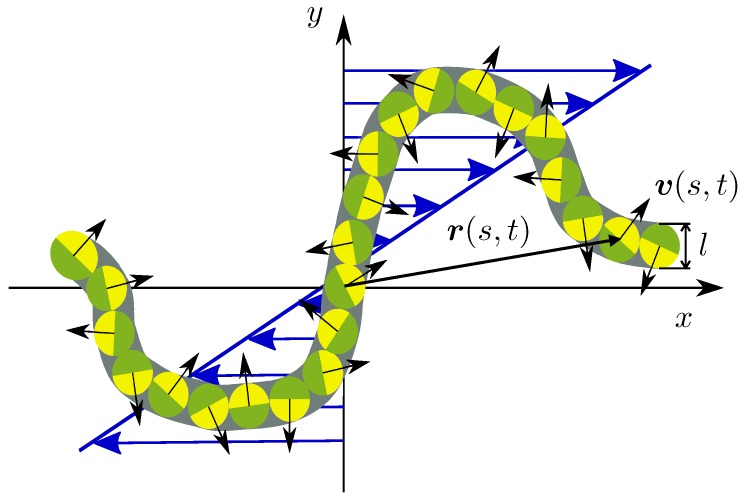
Illustration of the continuous semiflexible active polymer (ABPO) in shear flow. The arrows and colors indicate the orientation of the active velocity v(s,t).

The filament/polymer is described by the Gaussian semiflexible polymer model [75,76,77,78,79,80]. Thereby, the polymer of length *L* is considered as a continuous, differentiable space curve r(s,t), with the contour coordinate *s* (−L/2≤s≤L/2), and the time *t*. The activity is introduced by assigning an active velocity v(s,t) to every point r(s,t) (cf. Figure 1). The equation of motion of r(s,t) is then given by [52,53]

(1) $$\begin{array}{cc} \frac{\partial }{\partial t}r\left(s,t\right) & =v\left(s,t\right)+\frac{k_{B}T}{\gamma }\left(2\lambda \frac{\partial^{2}}{\partial s^{2}}r\left(s,t\right)-\epsilon \frac{\partial^{4}}{\partial s^{4}}r\left(s,t\right)\right)+\frac{1}{\gamma }\Gamma \left(s,t\right)+Kr\left(s,t\right) \end{array}$$

with the boundary conditions

(2) $$\begin{array}{c} {\left[2\lambda \frac{\partial }{\partial s}r\left(s,t\right)-\epsilon \frac{\partial^{3}}{\partial s^{3}}r\left(s,t\right)\right]}_{s=\pm L/2}=0,\;{\left[2\lambda_{0}\frac{\partial }{\partial s}r\left(s,t\right)\pm \epsilon \frac{\partial^{2}}{\partial s^{2}}r\left(s,t\right)\right]}_{s=\pm L/2}=0 \end{array}$$

The terms with the second and fourth derivative in Equation (1) account for the entropy elasticity and bending stiffness, respectively, Γ for thermal fluctuations, and Kr(s,t) for the shear flow, with the shear-rate tensor K. The Lagrangian multipliers $\lambda_{0}=\lambda \left(\pm L/2\right)$, λ(s), and ϵ are determined by constraints [81,82]. In general, this yields ϵ=3/4p and $\lambda_{0}=3/4$ for a polymer in three dimensions, where $p=1/2l_{p}$ in terms of the persistence length $l_{p}$ [75,82]. The Lagrangian multiplier λ, denoted as stretching coefficient, is determined in a mean-field manner via the global constraint [52,75,82]:

(3) $$\begin{array}{c} \int_{-L/2}^{L/2}\langle {\left(\frac{\partial r(s,t)}{\partial s}\right)}^{2}\rangle ds=L \end{array}$$

As discussed in Ref. [52], the active velocity v(s,t) can be considered as external colored noise experienced by the respective polymer site, the picture adopted here, or as intrinsic polymer property originating from self-propulsion. For an identical mathematical formulation, the active site has then to be described by an active Ornstein–Uhlenbeck particle (AOUP) [73,83]. However, also an active Brownian particle (ABP) can be considered, as long as only second moments of the active velocity correlation function are relevant [57]. In any case, the active velocity is described by a diffusive process—Brownian motion—either for the propulsion direction only (ABPs) [4,5,6,10,84,85], or for the individual Cartesian components, i.e., the magnitude of v is changing too (AOUPs) [4,52,73,83]. Independent of the details of the underlaying stochastic (active) processes, we denote our polymer as active Brownian polymer (ABPO). Hence, the active velocity, v(s,t), is described by a non-Markovian, but Gaussian stochastic process with zero mean and the second moments [52,53,73]

(4) $$\begin{array}{c} \langle v_{\alpha }\left(s,t\right)v_{\beta }\left(s^{\prime },t^{\prime }\right)\rangle =\frac{v_{0}^{2}l}{3}e^{-\gamma_{R}\left|t-t^{\prime }\right|}\delta \left(s-s^{\prime }\right)\delta_{\alpha \beta } \end{array}$$

i.e., the polymer is exposed to colored noise [52,53,73]. Here, $v_{0}$ is the propulsion velocity, the damping factor $\gamma_{R}$ can be related to the rotational diffusion coefficient $D_{R}$ of a spherical colloid in three dimensions via $\gamma_{R}=2D_{R}$, and $\alpha ,\beta \in \left\{x,y,z\right\}$. We introduce the length scale *l* in the continuum representation of a semiflexible polymer. Thereby, the ratio L/l can be interpreted as the number of uniformly distributed active sites along the polymer. In the flexible limit, we set p=1/l, which leads to the relation pL=L/l. The effect of the ratio L/l on the conformational properties in the absence of shear has briefly been addressed in Ref. [52]. The choice p=1/l is motivated by discrete bead-spring polymers, typically used in computer simulations [57,86], where every monomer is an ABP. The stochastic process Γ(s,t) of the translational motion is assumed to be stationary, Markovian, and Gaussian with zero mean and the second moments

(5) $$\begin{array}{c} \langle \Gamma_{\alpha }\left(s,t\right)\Gamma_{\beta }\left(s^{\prime },t^{\prime }\right)\rangle =2\gamma k_{B}T\delta_{\alpha \beta }\delta \left(s-s^{\prime }\right)\delta \left(t-t^{\prime }\right) \end{array}$$

where *T* is the temperature, $k_{B}$ the Boltzmann constant, and γ the translational friction coefficient per length. The latter is related with the translational, thermal diffusion coefficient $D_{T}$ via $D_{T}=k_{B}T/\gamma l$. Finally, shear is applied along the *x*-direction and the gradient along the *y*-direction of the Cartesian reference frame. Hence, the shear-rate tensor is given by $K_{xy}=\dot{\gamma }$, where $\dot{\gamma }$ is the shear rate.

### 2.2. Eigenfunction Expansion

The linear equation of motion (1) is solved by the eigenfunction expansion

(6) $$\begin{array}{c} r\left(s,t\right)=\sum_{n=0}^{\infty }\chi_{n}\left(t\right)\varphi_{n}\left(s\right) \end{array}$$

and an analogous representation of v and Γ, in terms of the eigenfunctions $\varphi_{n}$ of the eigenvalue equation [52,76]

(7) $$\begin{array}{c} \epsilon k_{B}T\frac{\partial^{4}}{\partial s^{4}}\varphi_{n}\left(s\right)-2\lambda k_{B}T\frac{\partial^{2}}{\partial s^{2}}\varphi_{n}\left(s\right)=\xi_{n}\varphi_{n}\left(s\right) \end{array}$$

The respective eigenfunctions are

(8) $$\begin{array}{cc} \varphi_{0} & =\sqrt{\frac{1}{L}}\; \end{array}$$

(9) $$\begin{array}{cc} \varphi_{n}\left(s\right) & =\sqrt{\frac{c_{n}}{L}}\left(\zeta_{n}^{\prime }\frac{\sinh(\zeta_{n}^{\prime }s)}{cosh(\zeta_{n}^{\prime }L/2)}+\zeta_{n}\frac{\sin(\zeta_{n}s)}{cos(\zeta_{n}L/2)}\right)\;,\;\forall n\;\mathrm{odd}\; \end{array}$$

(10) $$\begin{array}{cc} \varphi_{n}\left(s\right) & =\sqrt{\frac{c_{n}}{L}}\left(\zeta_{n}^{\prime }\frac{\cosh(\zeta_{n}^{\prime }s)}{sinh(\zeta_{n}^{\prime }L/2)}-\zeta_{n}\frac{\cos(\zeta_{n}s)}{sin(\zeta_{n}L/2)}\right)\;,\;\forall n\;\mathrm{even}\; \end{array}$$

with the relations between the wave numbers $\zeta_{n}$, $\zeta_{n}^{\prime }$, and the eigenvalues $\xi_{n}$ (n∈N)

(11) $$\begin{array}{c} \zeta_{n}^{\prime 2}-\zeta_{n}^{2}=\frac{2\lambda }{\epsilon }\;\;\;,\;\;\;\xi_{0}=0\;\;\;,\;\;\;\xi_{n}=k_{B}T\left(\epsilon \zeta_{n}^{4}+2\lambda \zeta_{n}^{2}\right)\;\; \end{array}$$

The eigenfunction $\varphi_{0}$ accounts for the polymer’s center of mass motion. The $c_{n}$s are normalization coefficients, and the wave numbers are determined by the boundary conditions (2).

Insertion of Equation (6) into Equation (1) yields the equation of motion for the mode amplitudes $\chi_{n}$,

(12) $$\begin{array}{c} \frac{\partial }{\partial t}\chi_{n\alpha }\left(t\right)=-\frac{1}{\tau_{n}}\chi_{n\alpha }\left(t\right)+v_{n\alpha }\left(t\right)+\frac{1}{\gamma }\Gamma_{n\alpha }\left(t\right)+\dot{\gamma }\chi_{ny}\delta_{x\alpha } \end{array}$$

with respective amplitudes $v_{n}\left(t\right)$ and $\Gamma_{n}\left(t\right)$ of the active velocity and stochastic force, and the relaxation times (n>0)

(13) $$\begin{array}{c} \tau_{n}=\frac{\gamma }{\xi_{n}}=\frac{\gamma }{k_{B}T\left(\epsilon \zeta_{n}^{4}+2\lambda \zeta_{n}^{2}\right)} \end{array}$$

The stationary-state solution of Equation (12) for n>0 is

(14) $$\begin{array}{c} \chi_{n\alpha }\left(t\right)=\int_{-\infty }^{t}dt^{\prime }e^{-\left(t-t^{\prime }\right)/\tau_{n}}\left[v_{n\alpha }\left(t^{\prime }\right)+\Gamma_{n\alpha }\left(t^{\prime }\right)+\dot{\gamma }\chi_{ny}\left(t^{\prime }\right)\delta_{x\alpha }\right] \end{array}$$

and for n=0

(15) $$\begin{array}{c} \chi_{0\alpha }\left(t\right)=\chi_{0\alpha }\left(0\right)+\int_{0}^{t}dt^{\prime }\left[v_{0\alpha }\left(t^{\prime }\right)+\Gamma_{0\alpha }\left(t^{\prime }\right)+\dot{\gamma }\chi_{0y}\left(t^{\prime }\right)\delta_{x\alpha }\right] \end{array}$$

### 2.3. Mode–Amplitude Correlation Functions

The time correlation functions of the mode amplitudes can be calculated straightforwardly by Equation (14), which yields $\langle \chi_{n}\left(t\right)\cdot \chi_{m}\left(t^{\prime }\right)\rangle =\delta_{nm}\langle \chi_{n}\left(t\right)\cdot \chi_{n}\left(t^{\prime }\right)\rangle$, with (n>0, t≥0)

(16) $$\begin{array}{cc} \langle \chi_{ny}\left(t\right)\chi_{ny}\left(0\right)\rangle = & \langle \chi_{nz}\left(t\right)\chi_{nz}\left(0\right)\rangle =\left(\frac{k_{B}T\tau_{n}}{\gamma }-\frac{v_{0}^{2}l\gamma_{R}\tau_{n}^{3}}{3\left(1-{\left(\gamma_{R}\tau_{n}\right)}^{2}\right)}\right)e^{-t/\tau_{n}}+\frac{v_{0}^{2}l\tau_{n}^{2}}{3\left(1-{\left(\gamma_{R}\tau_{n}\right)}^{2}\right)}e^{-\gamma_{R}t} \end{array}$$

(17) $$\begin{array}{cc} \langle \chi_{nx}\left(t\right)\chi_{nx}\left(0\right)\rangle = & \langle \chi_{ny}\left(t\right)\chi_{ny}\left(0\right)\rangle +\frac{{\dot{\gamma }}^{2}v_{0}^{2}l\tau_{n}^{4}}{3{\left(1-{\left(\gamma_{R}\tau_{n}\right)}^{2}\right)}^{2}}e^{-\gamma_{R}t}\\ + & \left[\frac{{\dot{\gamma }}^{2}\tau_{n}^{2}}{2}\left(\frac{k_{B}T\tau_{n}}{\gamma }-\frac{v_{0}^{2}l\gamma_{R}\tau_{n}^{3}}{3\left(1-{\left(\gamma_{R}\tau_{n}\right)}^{2}\right)}\right)\left(1+\frac{t}{\tau_{n}}\right)-\frac{{\dot{\gamma }}^{2}\gamma_{R}v_{0}^{2}l\tau_{n}^{5}}{3{\left(1-{\left(\gamma_{R}\tau_{n}\right)}^{2}\right)}^{2}}\right]e^{-t/\tau_{n}} \end{array}$$

(18) $$\begin{array}{cc} \langle \chi_{nx}\left(t\right)\chi_{ny}\left(0\right)\rangle = & \frac{\dot{\gamma }v_{0}^{2}l\tau_{n}^{3}}{3{\left(1-(\gamma_{R}\tau_{n}\right)}^{2})\left(1-\gamma_{R}\tau_{n}\right)}e^{-\gamma_{R}t}\\ + & \left[\frac{\dot{\gamma }\tau_{n}}{2}\left(\frac{k_{B}T\tau_{n}}{\gamma }-\frac{v_{0}^{2}l\gamma_{R}\tau_{n}^{3}}{3\left(1-{\left(\gamma_{R}\tau_{n}\right)}^{2}\right)}\right)\left(1+\frac{2t}{\tau_{n}}\right)-\frac{2\dot{\gamma }\gamma_{R}v_{0}^{2}l\tau_{n}^{4}}{3{\left(1-{\left(\gamma_{R}\tau_{n}\right)}^{2}\right)}^{2}}\right]e^{-t/\tau_{n}} \end{array}$$

### 2.4. Inextensibility and Stretching Coefficient λ

The inextensibility constraint (3), together with the eigenfunction expansion and the correlation functions (16)–(18), leads to the equation

(19) $$\begin{array}{c} \sum_{n=1}^{\infty }\langle \chi_{n}^{2}\rangle \phi_{n}=1 \end{array}$$

to determine λ, where $\phi_{n}=\int_{-L/2}^{L/2}{\left(\partial \varphi_{n}\left(s\right)/\partial s\right)}^{2}ds/L$ and

(20) $$\begin{array}{c} \langle \chi_{n}^{2}\rangle =\frac{3k_{B}T}{\gamma }\tau_{n}+\frac{v_{0}^{2}l}{1+\gamma_{R}\tau_{n}}\tau_{n}^{2}+{\dot{\gamma }}^{2}\frac{k_{B}T}{2\gamma }\tau_{n}^{3}+{\dot{\gamma }}^{2}\frac{v_{0}^{2}l\left(2+\gamma_{R}\tau_{n}\right)}{6{\left(1+\gamma_{R}\tau_{n}\right)}^{2}}\tau_{n}^{4} \end{array}$$

In general, Equation (19) has to be solved numerically. However, the sum over the mode numbers can be evaluated in the limit pL→∞, or even for moderate pL, for larger activities Pe≫1 [53], due to the dominance of the stretching modes in these limits, i.e., $\zeta_{n}=n\pi /L$ and $\tau_{n}=\gamma L^{2}/3k_{B}T\pi^{2}p\mu n^{2}=\tau_{R}/\mu n^{2}$, where $\tau_{R}=\gamma L^{2}/3k_{B}T\pi^{2}p$ is the Rouse relaxation time of the passive polymer [76,87]. Here, the Péclet number Pe and other relevant dimensionless quantities, such as the Weissenberg number Wi, the ratio between the translational and rotational diffusion coefficient Δ, and the scaled stretching coefficient μ, in terms of the value at equilibrium, 3p/2, are introduced as

(21) $$\begin{array}{c} Pe=\frac{v_{0}}{D_{R}l},\;Wi=\dot{\gamma }\tau_{0},\;\Delta =\frac{D_{T}}{l^{2}D_{R}},\;\mu =\frac{2\lambda }{3p} \end{array}$$

Thereby, $\tau_{0}=\tau_{1}\left(Pe,Wi=0\right)$ is the longest polymer relaxation time at zero shear but in the presence of activity. Combined with results from Refs. [52,88], we find from Equation (19)

(22) $$\begin{array}{cc} \frac{1}{\sqrt{\mu }}\coth\left(2pL\sqrt{\mu }\right)-\frac{1}{2pL\mu } & +\frac{Pe^{2}}{6\mu \Delta }\left[\sqrt{\frac{\mu }{1+6\mu^{2}p^{3}l^{3}\Delta }}\coth\left(2pL\sqrt{\frac{\mu }{1+6\mu^{2}p^{3}l^{3}\Delta }}\right)-\frac{1}{2pL}\right]\\ & +\frac{Wi^{2}\pi^{4}}{540pL}\frac{\mu_{0}^{2}}{\mu^{3}}+\frac{Wi^{2}Pe^{2}L^{3}\left(2+2L^{3}/3\pi^{2}l^{3}\Delta pL\mu \right)}{54\pi^{2}\Delta^{2}{\left(pL\right)}^{2}l^{3}{\left(1+2L^{3}/3\pi^{2}l^{3}\Delta pL\mu \right)}^{2}}\frac{\mu_{0}^{2}}{\mu^{4}}=1 \end{array}$$

where for the evaluation of the term proportional to $Wi^{2}Pe^{2}∼{\dot{\gamma }}^{2}v_{0}^{2}$ only the first mode, n=1, has been taken into account; the deviation to the full sum is below 3% for all Pe,pL, and Wi. The last term on the left-hand side reflects the coupling between activity and shear flow.

The following asymptotic dependencies for the stretching coefficient μ are obtained:

(i) Passive semiflexible polymer in shear flow, i.e., Pe=0 (for details, cf. Ref. [88])
  - For pL≫1 and μ≫1
    (23) $$\begin{array}{c} \mu^{3}-\mu^{5/2}-\frac{\pi^{4}Wi^{2}}{540pL}=0,\;\overset{Wi\gg 1}{⟹}\mu =Wi^{2/3}{\left(\frac{\pi^{4}}{540pL}\right)}^{1/3} \end{array}$$
  - For pL<1 and μ≫1
    (24) $$\begin{array}{c} \mu =\frac{Wi^{2/3}}{pL}{\left(\frac{4}{15}\right)}^{1/3} \end{array}$$
(ii) Active flexible polymer at weak shear flow, i.e., Wi≪1, pL≫1, and $\tau_{n}=\tau_{R}/\mu_{0}n^{2}$ [52,53]. For later use, we denote the Lagrangian multiplier at Wi=0 and Pe>0 by $\mu_{0}$
  - For 1≪Pe<∞ and $\mu_{0}\to \infty$
    (25) $$\begin{array}{c} \mu_{0}=\frac{Pe^{4/3}}{pL}\frac{L}{6l\Delta } \end{array}$$
  - For Pe→∞, i.e., $\mu_{0}\to \infty$
    (26) $$\begin{array}{c} \mu_{0}=\frac{Pe}{pL\Delta }\sqrt{\frac{L^{3}}{54l^{3}}} \end{array}$$
(iii) Active flexible polymer in shear flow, $\tau_{n}=\tau_{R}/\mu n^{2}$,
  - For 1<Pe,Wi<∞, $L^{3}/3\pi l^{3}pL\Delta \mu \gg 1$, and pL≫1 (with Equation (25))
    (27) $$\begin{array}{c} \mu =Wi^{2/3}\mu_{0}^{2/3}{\left(\frac{Pe^{2}}{36\Delta pL}\right)}^{1/3}=Wi^{2/3}\frac{Pe^{14/9}}{pL\Delta }{\left(\frac{L}{36l}\right)}^{2/3} \end{array}$$
  - For Pe→∞, i.e., $\mu_{0}=Pe\sqrt{L^{3}/54l^{3}}/pL\Delta \to \infty$
    (28) $$\begin{array}{c} \mu =\mu_{0}\frac{1}{\sqrt{2}}\sqrt{1+\sqrt{1+\frac{8Wi^{2}}{\pi^{2}}}}\;\overset{Wi\gg 1}{⟶}\;Wi^{1/2}\frac{Pe}{pL\Delta }\sqrt{\frac{\sqrt{2}L^{3}}{54\pi l^{3}}} \end{array}$$
  Hence, in the limit Wi→∞, μ exhibits a crossover from a $\mu ∼Wi^{2/3}$ dependence for 1≪Pe<∞ to a dependence $\mu ∼Wi^{1/2}$ for Pe→∞. The latter characteristics are different from the passive case and are a consequence of the coupling between activity and shear flow.

**Figure 2:**
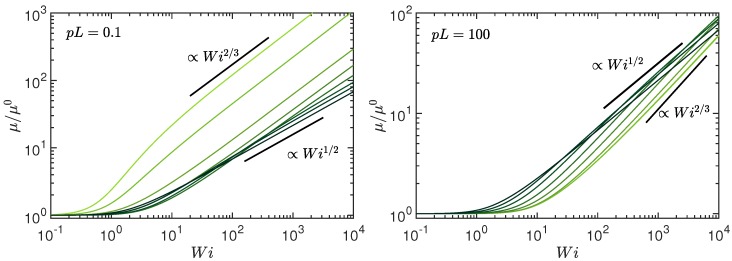
Stretching coefficient μ=2λ/3p normalized by the value $\mu_{0}$ of the active, non-sheared system as function of the Weissenberg number Wi for the Péclet numbers $Pe=0,\;0.6,\;3,\;10,\;30,\;10^{2},3\times 10^{2},$ and ∞ (bright to dark color); (**left**) $pL=L/2l_{p}=0.1$ (stiff) and (**right**) $pL=10^{2}$ (flexible polymer). The number of active sites is $L/l=10^{2}$ and the diffusion coefficient ratio Δ=0.3.

**Figure 3:**
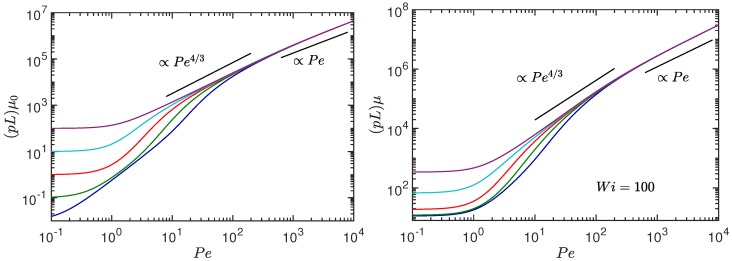
Stretching coefficient μ=2λ/3p as a function of the Péclet number Pe for the stiffness $pL=10^{-2},\;10^{-1},\;10^{0},\;10^{1},$ and $10^{2}$ (bottom to top). The Weissenberg numbers are (**left**) Wi=0 [52] and (**right**) $Wi=10^{2}$. The number of active sites is $L/l=10^{2}$ and Δ=0.3.

The full numerical solution for the stretching coefficient μ is presented in Figure 2 as a function of the Weissenberg number and for various activities. We set $L/l=10^{2}$ for the number of active sites. Hence, when changing pL, we change the persistence length $l_{p}=1/2p$ at a fixed contour length in order to maintain the active-site density. As illustrated for pL=0.1 and $pL=10^{2}$, μ exhibits a crossover from a dependence $\mu ∼Wi^{2/3}$ at low Pe to the relation $\mu ∼Wi^{1/2}$ for Pe→∞ and sufficiently large Wi, in agreement with the theoretical limits, Equations (27) and (28). We like to emphasize that $\mu /\mu_{0}$ approaches the asymptotic dependence

(29) $$\begin{array}{c} \frac{\mu }{\mu_{0}}=\sqrt{Wi}\sqrt{\frac{\sqrt{2}}{\pi }} \end{array}$$

for Pe≫1. Hence, a universal, activity- and pL-independent behavior is predicted. For completeness, Figure 3 illustrates the dependence of μ on the Péclet number for the Weissenberg numbers Wi=0 (left) and $Wi=10^{2}$ (right). The predicted power-law dependencies (Equation (25) for Wi<1, and Equation (28) for Pe≫1 and Wi≪Pe) and scaling with respect to pL are recovered. Figure 2 shows a shift of the crossover from the $Wi^{2/3}$ to the $Wi^{1/2}$ dependence towards smaller Wi with increasing Pe. This crossover strongly depends on L/l, and shifts to larger Wi with increasing L/l. Hence, for a larger number of active sites, no crossover could be observed anymore for suitable Weissenberg numbers. On the contrary, for a smaller number L/l, the crossover appears already at smaller Wi. In the extreme case of L/l→1, a behavior similar to an active dumbbell in shear flow appears [51].

## 3. Dynamics and Conformations

### 3.1. Relaxation Times

The relaxation times (13) depend on the shear rate (Wi), activity (Pe), and persistence length (*p*) via the stretching coefficient λ=2μ/3p. In the limit of a highly flexible polymer, the relaxation time is $\tau_{n}=\tau_{R}/\left(\mu n^{2}\right)$. Hence, the mode-number dependence of $\tau_{n}$ is unaffected by the nonequilibrium character of the dynamics. However, the presence of μ indicates the fundamental importance to account for the inextensibility of the polymer. Since μ≥1 is a monotonically increasing function of Pe and Wi, activity and shear flow always accelerate the relaxation process and the relaxation times become shorter [35,53].

**Figure 4:**
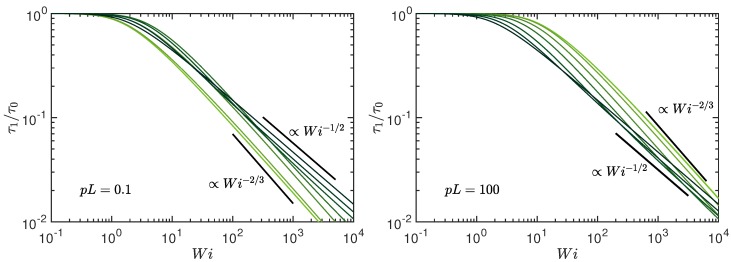
Longest polymer relaxation time $\tau_{1}$ normalized by the longest relaxation time $\tau_{0}$ of the active, non-sheared system (Wi=0) as function of the Weissenberg number Wi for the Péclet numbers $Pe=0,\;0.6,\;3,\;10,\;30,\;10^{2},3\times 10^{2},$ and ∞ (bright to dark color); (**left**) pL=0.1 and (**right**) $pL=10^{2}$. The number of active sites is $L/l=10^{2}$ and Δ=0.3.

Figure 4 displays the numerically obtained longest relaxation time as a function of the Weissenberg number for various Pe and the stiffness pL=0.1 (stiff) and $pL=10^{2}$ (flexible polymer). For pL≪1 and small Péclet numbers (Pe≲1), the relaxation time $\tau_{1}$, corresponding to the rotation relaxation time of a rigid polymer, dominates over all other (bending) relaxation times [53,76]. Hence, the relaxation times of Figure 4 (left) are not simply proportional to $\mu^{-1}$ in this limit. However, with increasing Pe, bending contributions gradually vanish and the asymptotic dependence $\tau_{1}/\tau_{0}=\mu_{0}/\mu$ is assumed. According to Equation (29), the ratio $\tau_{1}/\tau_{0}$ is then independent of Pe and pL. In Figure 4 (right) for flexible polymers, bending modes are negligibly small and the relation $\tau_{1}∼1/\mu$ applies for all Pe. Consequently, $\tau_{1}$ exhibits the power-law dependencies of Equations (23), (27), and (28).

We like to emphasize that the shear-rate dependency $\tau_{1}∼1/\sqrt{Wi}$ is a consequence of the activity of the polymer and emerges from the coupling of activity and shear (cf. Equation (22)). The passive polymer under shear exhibits the dependence $\tau_{1}∼Wi^{-2/3}$, which we find for small Pe also for the active polymer. A dumbbell of active monomers exhibits a similar coupling of activity and shear and, correspondingly, shows a comparable crossover of the relaxation times [88].

**Figure 5:**
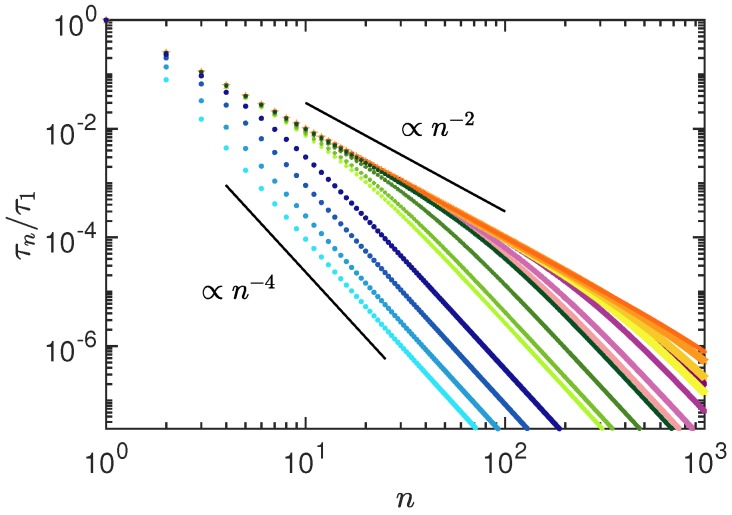
Mode-number dependence of the relaxation times $\tau_{n}$ normalized by the longest relaxation time $\tau_{1}$ for the Péclet numbers $Pe=0,\;10^{1},\;10^{2}$, and $10^{3}$ (different colors and symbols; from left to right), and the Weissenberg numbers $Wi=0,\;10^{1},\;10^{2}$, and $10^{3}$ (different tone, bright to dark, for every color). The persistence length is pL=1 and $L/l=10^{2}$.

Figure 5 illustrates the mode-number dependence of the relaxation times for various activities and shear rates. For semiflexible polymers, activity and shear flow modify the relaxation behavior because stretching modes ($n^{2}$) dominate over bending modes ($n^{4}$) with increasing activity and flow strength. Bending stiffness remains dominant at larger mode numbers. Activity as well as flow induce a transition from semiflexible to flexible polymer behavior, which extends to smaller and smaller length scales with increasing Pe and Wi.

### 3.2. Radius of Gyration

The polymer conformations are characterized by the radius of gyration tensor *G*, with the components

(30) $$\begin{array}{c} G_{\alpha \beta }=\frac{1}{L}\int_{-L/2}^{L/2}\langle \left(r_{\alpha }\left(s\right)-r_{cm\alpha }\right)\left(r_{\beta }\left(s\right)-r_{cm\beta }\right)\rangle ds \end{array}$$

where $r_{cm}$ is the center-of-mass position of the polymer. Insertion of the eigenfunction expansion (6) yields

(31) $$\begin{array}{c} G_{\alpha \beta }=\frac{1}{L}\sum_{n=1}^{\infty }\langle \chi_{n\alpha }\left(t\right)\chi_{n\beta }\left(t\right)\rangle \end{array}$$

in terms of the mode-amplitude correlation functions $\langle \chi_{n\alpha }\chi_{n\beta }\rangle$ (Equations (16)–(18)).

**Figure 6:**
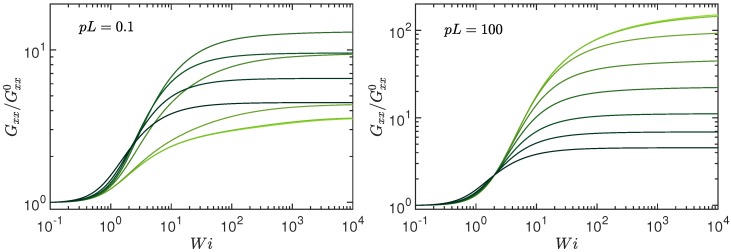
Radius of gyration-tensor component $G_{xx}$ along the flow direction normalized by the value $G_{xx}^{0}$ at zero shear as function of the Weissenberg number Wi for the Péclet numbers $Pe=0,\;0.6,\;3,\;10,\;30,\;10^{2},3\times 10^{2},$ and ∞ (bright to dark color); (**left**) pL=0.1 and (**right**) $pL=10^{2}$. The number of active sites is $L/l=10^{2}$ and Δ=0.3.

Figure 6 depicts the radius of gyration-tensor component $G_{xx}$ along the flow direction for rather stiff (pL=0.1) and highly flexible ($pL=10^{2}$) polymers. Note that only the excess deformation due to shear is shown. Activity leads to additional conformational changes, which are included in $G_{xx}^{0}=G_{xx}\left(Wi=0\right)$. As for passive semiflexible polymers, shear leads to an extension and alignment along the flow direction, which saturates at large shear rates because of the finite polymer contour length [88,89]. The actual asymptotic stretching for Wi→∞ depends on the activity. At pL≫1, the asymptotic limits are $G_{xx}^{\infty }/G_{xx}^{0}=12pL/7$ for Pe=0 [88], and $G_{xx}^{\infty }=L^{2}\pi^{6}/9450$, $G_{xx}^{0}=L^{2}/45$, hence $G_{xx}^{\infty }/G_{xx}^{0}=\pi^{6}/210$ for Pe→∞. It is noteworthy that the latter limit is independent of pL, i.e., it applies for every stiffness, and the same asymptotic behavior is displayed in Figure 6 (left) and (right). Shear flow leads to an additional stretching of the active polymer, particularly for Pe→∞, and not simply to an orientational alignment as for a rod, where $G_{xx}^{\infty }/G_{xx}^{0}=3$ for Wi≫1 [88], since $\pi^{6}/210\approx 4.6>3$. However, the difference of the asymptotic values for Pe=0 and Pe→∞, respectively, can be substantial, since $G_{xx}^{\infty }$ of the passive system depends on polymer length. The polymer pre-stretching by activity reduces the possible stretching by shear. The asymptotic limits for pL→0 at Pe=0 are $G_{xx}^{\infty }=10L^{2}/105$ and $G_{xx}^{0}=L^{2}/36$, hence, $G_{xx}^{\infty }/G_{xx}^{0}=24/7$, in agreement with Figure 6 (left). Note that $G_{xx}/G_{xx}^{0}$ depends non-monotonically on the Péclet number at small pL. The ratio $G_{xx}/G_{xx}^{0}$ increases with increasing Pe at small Pe and decreases again for Pe≫1 (cf. Figure 6 (left)). In contrast, $G_{xx}/G_{xx}^{0}$ decrease monotonically with increasing Pe at large pL (cf. Figure 6 (right)). In any case, shear leads to an alignment and additional stretching even in the limit of very large activity.

**Figure 7:**
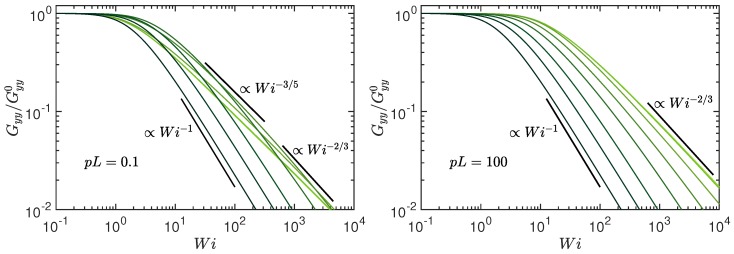
Radius of gyration-tensor component $G_{yy}$ along the gradient direction normalized by the value $G_{yy}^{0}$ at zero shear as function of the Weissenberg number Wi for the Péclet numbers $Pe=0,\;0.6,\;3,\;10,\;30,\;10^{2},3\times 10^{2},$ and ∞ (bright to dark color); (**left**) pL=0.1 and (**right**) $pL=10^{2}$. The number of active sites is $L/l=10^{2}$ and Δ=0.3.

The radius of gyration-tensor component along the gradient direction is displayed in Figure 7. Note that $G_{yy}\equiv G_{zz}$. Consistent with the extension in the flow direction, a polymer shrinks in the transverse direction. We find the asymptotic dependencies for Wi→∞ and Pe=0, $G_{yy}/G_{yy}^{0}=\sqrt[{3}]{30}Wi^{-2/3}$ for pL→0, and $G_{yy}/G_{yy}^{0}=\sqrt[{3}]{540pL/\pi^{4}}Wi^{-2/3}$ for pL≫1 . In the limit Pe→∞, $G_{yy}/G_{yy}^{0}=\pi /\left(\sqrt{2}Wi\right)$ independent of pL. Again, the latter dependence is specific for active systems, since passive polymers typically show a weaker dependence on the Weissenberg number [88,89].

### 3.3. Alignment

Anisotropic objects in shear flow are preferentially aligned along the flow direction [88,89]. We characterize the extent of alignment by the angle $\chi_{G}$ between the eigenvector of the gyration tensor with the largest eigenvalue and the flow direction. The alignment angle is conveniently obtained from the relation

(32) $$\begin{array}{c} \tan\left(2\chi_{G}\right)=\frac{2G_{xy}}{G_{xx}-G_{yy}}=\frac{\sum_{n=1}^{\infty }\langle \chi_{nx}\chi_{ny}\rangle }{\sum_{n=1}^{\infty }\langle \chi_{nx}^{2}\rangle -\langle \chi_{ny}^{2}\rangle } \end{array}$$

where

(33) $$\begin{array}{c} \langle \chi_{nx}\chi_{ny}\rangle =\frac{\dot{\gamma }k_{B}T}{2\gamma }\tau_{n}^{2}+\frac{\dot{\gamma }v_{0}^{2}l\left(2+\gamma_{R}\tau_{n}\right)}{6{\left(1+\gamma_{R}\tau_{n}\right)}^{2}}\tau_{n}^{3} \end{array}$$

In the asymptotic limit μ≫1, i.e., $\tau_{n}\approx \tau_{R}/n^{2}\mu$, this expression reduces to [51]

(34) $$\begin{array}{c} \tan\left(2\chi_{G}\right)=\frac{2\mu }{Wi\mu_{0}} \end{array}$$

**Figure 8:**
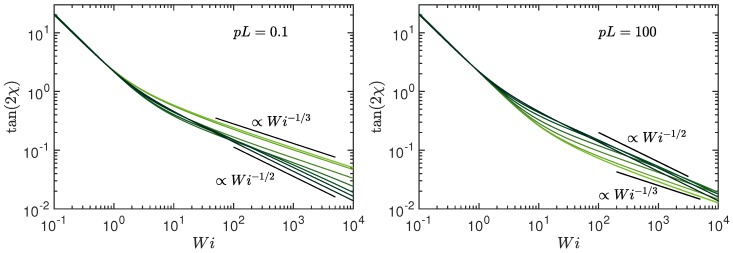
Shear-induced polymer alignment, characterized by the angle $\chi_{G}$ between the eigenvector of the gyration tensor with the largest eigenvalue and the flow direction, as function of the Weissenberg number Wi. The Péclet numbers are $Pe=0,\;0.6,\;3,\;10,\;30,\;10^{2},3\times 10^{2},$ and ∞ (bright to dark color); (**left**) pL=0.1 and (**right**) $pL=10^{2}$. The number of active sites is $L/l=10^{2}$ and Δ=0.3.

Hence, we obtain the asymptotic dependence $\tan\left(2\chi_{G}\right)∼Wi^{-1/3}$ for Pe→0 and $\tan\left(2\chi_{G}\right)∼Wi^{-1/2}$ for Pe→∞, respectively. The various regimes are displayed in Figure 8. For Wi<1, the stretching coefficient is approximately unity and $\tan(2\chi_{G})$ decreases as $Wi^{-1}$. For large Weissenberg numbers, the shear-rate dependence of μ becomes important and changes the Wi dependence to $\tan\left(2\chi \right)∼Wi^{-1/3}$ for Pe≪1 and to $\tan\left(2\chi \right)∼Wi^{-1/2}$ for Pe≫1.

## 4. Rheology: Viscosity

The polymer contribution $\eta_{p}$ to the viscosity of a dilute solution follows from the virial expression of the stress tensor

(35) $$\begin{array}{c} \sigma_{xy}=-\rho \int_{-L/2}^{L/2}\langle F_{x}\left(s\right)r_{y}\left(s\right)\rangle ds \end{array}$$

via $\eta_{p}=\sigma_{xy}/\dot{\gamma }$, where *F* is the intramolecular force of Equation (1) and ρ the polymer concentration. The active force, γv(s,t), does not contribute to the stress tensor. Evaluation of the average in Equation (35) yields

(36) $$\begin{array}{c} \eta_{p}=\frac{\rho k_{B}T\gamma }{\dot{\gamma }}\sum_{n=1}^{\infty }\frac{1}{\tau_{n}}\langle \chi_{nx}\chi_{ny}\rangle =\frac{\rho k_{B}T}{2}\sum_{n=1}^{\infty }\left[\tau_{n}+\frac{\gamma v_{0}^{2}l\left(2+\gamma_{R}\tau_{n}\right)}{3k_{B}T{\left(1+\gamma_{R}\tau_{n}\right)}^{2}}\tau_{n}^{2}\right] \end{array}$$

which depends via the stretching coefficient μ on the shear rate.

**Figure 9:**
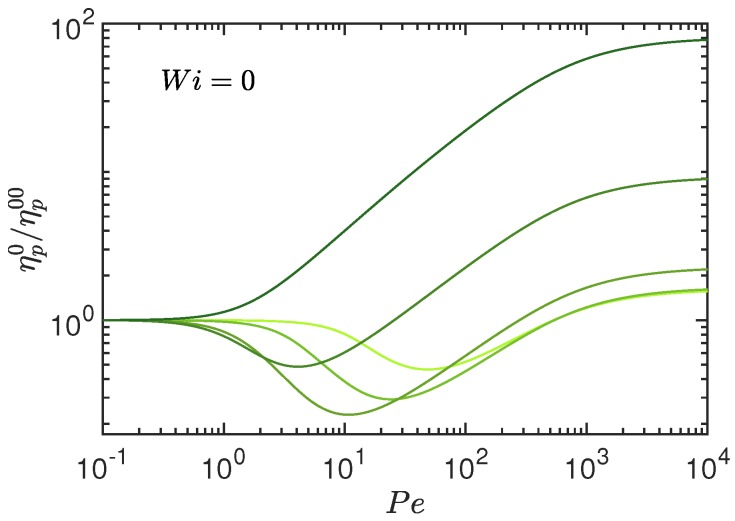
Zero-shear viscosity $\eta_{p}^{0}$ normalized by the zero-shear viscosity $\eta_{p}^{00}$ of a passive polymer as function of the Péclet number Pe for the polymer stiffness pL=100,10,1,0.1, and 0.01 (top to bottom at $Pe=10^{4}$, dark to bright color). The number of active sites is $L/l=10^{2}$ and Δ=0.3.

The zero-shear viscosity $\eta_{p}^{0}$ follows from Equation (36) via the stretching coefficient $\mu_{0}$. Its dependence on Pe is shown in Figure 9. The viscosity $\eta_{p}^{00}$ at zero shear and zero Péclet number is given by $\eta_{p}^{00}=\rho k_{B}T\pi^{2}\tau_{R}/12$ for pL≫1, and by $\eta_{p}^{00}=\gamma L^{3}\rho /72$ for Pe→0. In the latter case, the first mode, $\tau_{1}=\gamma L^{3}/36k_{B}T$, describing the rotational motion of the rodlike polymer, dominates the sum over the relaxation times [76]. For flexible polymers, where pL≫1, the zero-shear viscosity increases monotonically with increasing Pe, and saturates at $\eta_{p}^{0}/\eta_{p}^{00}=4pL/5$ in the limit Pe→∞. Thereby, the viscosity increase, associated with the monotonic swelling of the polymer with increasing activity [52], is substantial because $\eta_{p}^{0}/\eta_{p}^{00}∼pL$. With increasing stiffness, the activity-induced polymer shrinkage (cf. Ref. [52]) implies a decrease in $\eta_{p}^{0}$, followed by an increase due to a reswelling of the polymer for Pe→∞, and the asymptotic value $\eta_{p}^{0}/\eta_{p}^{00}=8/5$ is assumed. Here, the activity dependence of $\eta_{p}^{0}$ is significantly smaller than for flexible polymers, and reduces to a factor below two in the rod limit.

**Figure 10:**
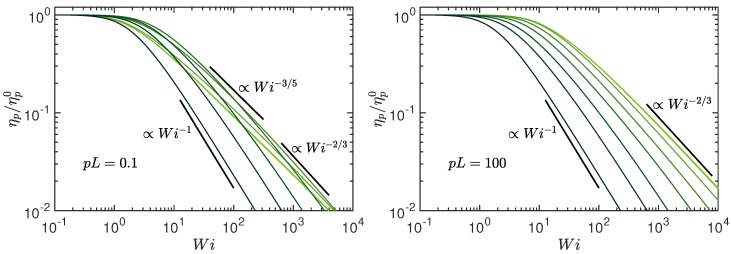
Shear viscosity $\eta_{p}$ normalized by the viscosity $\eta_{p}^{0}$ of a non-sheared, active polymer as function of the Weissenberg number Wi for the Péclet numbers $Pe=0,\;0.6,\;3,\;10,\;30,\;10^{2},3\times 10^{2},$ and ∞ (bright to dark color); (**left**) pL=0.1 and (**right**) $pL=10^{2}$. The number of active sites is $L/l=10^{2}$ and Δ=0.3.

The shear-rate dependence of the viscosity $\eta_{p}$, normalized by $\eta_{p}^{0}$, is displayed in Figure 10 for various Péclet numbers. Independent of persistence length and activity, the polymers exhibit shear thinning. However, the dependence on the Weissenberg number is strongly affected by the activity. The behavior of passive semiflexible polymers, where Pe = 0, has been discussed theoretically in Ref. [88]. For such polymers, the viscosity exhibits the asymptotic dependencies for Wi→∞: $\eta_{p}/\eta_{p}^{0}={\left(540pL/\pi^{4}\right)}^{1/3}Wi^{-2/3}$ for pL≫1 and $\eta_{p}/\eta_{p}^{0}={\left(30\right)}^{1/3}Wi^{-2/3}$ for pL<1. In fact, for large stiffness, $pL=L/2l_{p}<1$, there is a cross-over regime with an approximate power-law drop of $\eta_{p}∼Wi^{-3/5}$ as indicated in Figure 10 (left). Here, both bending and stretching modes contribute with a Weissenberg number-dependent weight. Activity substantially changes the shear-thinning behavior, and, with increasing Pe, the ratio $\eta_{p}/\eta_{p}^{0}$ decreases faster with increasing shear rate. From Equation (36), we obtain the relation

(37) $$\begin{array}{c} \frac{\eta_{p}}{\eta_{p}^{0}}=\frac{\mu_{0}^{2}}{\mu^{2}}\overset{Wi\to \infty }{=}\frac{\pi }{\sqrt{2}Wi} \end{array}$$

in the limit Pe→∞, which is independent of pL. Hence, activity enhances shear thinning considerably.

Shear thinning of passive polymers, where Pe=0, has intensively been studied experimentally [90,91], theoretically [88], and by simulations [89,92,93,94,95,96,97]. Specifically, measurements on DNA molecules provided insight into the behavior of individual polymers [91]. These experiments and simulations often predicted a power-law decay of the viscosity in the shear-thinning regime, with exponents in the range 1/2 to 2/3. The spread is partially explained by the very broad crossover regime between the zero-shear viscosity and the asymptotic dependence for Wi→∞. In any case, activity is predicted to lead to a significantly stronger shear-thinning effect.

## 5. Discussion

The coupling between shear flow and activity, as is visible in the correlation functions (16)–(18), determines the characteristics of an ABPO in shear flow. The shear-rate dependence of all properties—conformational, dynamical, and rheological—are modified by activity. Thereby, the determining factor is the polymer inextensibility, which is reflected in the activity and shear-rate dependence of the stretching coefficient λ=3pμ/2 in our coarse-grained description. In particular, the asymptotic behavior for Pe,Wi→∞ is naturally governed by inextensibility. As far as the dynamics is concerned, we find a weaker variation of the relaxation times with shear rate at large activities compared to a passive polymer, with the longest relaxation time $\tau_{1}$ changing from a $\tau_{1}∼Wi^{-2/3}$ dependence of a passive polymer to a $\tau_{1}∼Wi^{-1/2}$ decay for Pe≫1. In turn, this results in a change of the shrinkage of the radius of gyration components $G_{yy}=G_{zz}$ from a $Wi^{-2/3}$ to a $Wi^{-1}$ dependence, a similar change for the viscosity $\eta_{p}$, and a change of the alignment from a $Wi^{-1/3}$ to a $Wi^{-1/2}$ dependence with increasing Wi at Pe≫1. As has already been discussed in Ref. [52], flexible and semiflexible ABPO show the same activity-induced swelling behavior for Pe≫1, independent of pL. Consequently, a universal shear-flow behavior is obtained in that limit. For all conformational ($G_{xx},\;G_{yy},\;\tan\left(2\chi_{G}\right)$), dynamical ($\tau_{1}$), and rheological ($\eta_{p}$) properties, universal curves are obtained, with shear-rate dependencies differing from those of a passive system. The behavior originates from the dominance of the flexible modes ($n^{2}$) in the relaxation behavior for all stiffness caused by activity.

Active dumbbells already exhibit various of the discussed shear-induced characteristics [51]. However, the polymer nature, with the many more internal degrees of freedom, provides additional features and means of controlling active properties. Specifically, the number of active sites, L/l, is important. As our study shows, the crossover from the power laws valid for passive polymers to those of an ABPO at P≫1 depends crucially on L/l. With increasing L/l, the power laws for Pe≫1 appear at much larger Weissenberg numbers only. Depending on the size of the polymer, the Weissenberg numbers of the crossover could exceed experimentally accessible values. For computer simulations of an ABPO described as bead-spring polymer [57,86], this aspect is of minor concern because typically every monomer is considered as an ABP and not too long polymers are studied.

## 6. Conclusions

We have presented analytical results for active semiflexible polymers under shear flow. The Gaussian semiflexible polymer model is adopted, which takes into account the polymer inextensibility in a mean-field manner by a constraint for the contour length [75,80,82]. Activity is modeled as a colored noise force with an exponential temporal correlation. The linearity of the equation of motion, even in the presence of shear flow, allows for its analytical solution.

We have calculated the relaxation times, deformation, alignment, and viscosity as a function of shear rate. Each of these quantities shows a strong dependence on shear rate. Thereby, activity affects the shear response. An important aspect of a polymer in shear flow is its stretching and alignment along the flow direction, and its shrinkage transverse to it [88,89,95]. Activity enhances these aspects for flexible polymers. Semiflexible polymers show a nonmonotonic deformation behavior as a result of an activity-induced shrinkage at moderate Péclet numbers and a swelling at larger Pe, where the latter is similar to that of flexible polymers at the same Pe [52]. The activity-induced preference in alignment leads to a more pronounced shear thinning of highly active polymers, i.e., activity enhances shear thinning. All polymers exhibit the same shear-rate dependence in the limit Pe→∞, and, consequently, a universal behavior is obtained.

The active polymer relaxation behavior is governed by two processes, namely the diffusive dynamics of the active velocity, characterized by $\gamma_{R}$, and the relaxation times of the polymer. It remains to be analyzed how these competing processes determine the overall relaxation dynamics, e.g., of the end-to-end vector, and diffusion of the ABPO in the presence of shear flow. 

References
